# Intensity of Intrathecal Total IgG Synthesis in Multiple Sclerosis Correlates with the Degree of Pleocytosis, Diversity of Intrathecal Antiviral Antibody Specificities, and Female Sex

**DOI:** 10.3390/antib13040102

**Published:** 2024-12-12

**Authors:** Benjamin Vlad, Marc Hilty, Stephan Neidhart, Klara Asplund Högelin, Mario Ziegler, Mohsen Khademi, Andreas Lutterotti, Axel Regeniter, Roland Martin, Faiez Al Nimer, Ilijas Jelcic

**Affiliations:** 1 Neuroimmunology and Multiple Sclerosis Research Section, Department of Neurology, University Hospital Zurich, 8091 Zurich, Switzerland; benjamindaniel.vlad@med.uni-jena.de (B.V.); marc.hilty@hirslanden.ch (M.H.); stephan.neidhart@kliniklengg.ch (S.N.); mario.ziegler@usz.ch (M.Z.); andreas.lutterotti@bmg-swiss.ch (A.L.); roland.martin@uzh.ch (R.M.); 2 Faculty of Medicine, University of Zurich, 8006 Zurich, Switzerland; 3 Department of Neurology, University Hospital Jena, 07749 Jena, Germany; 4 Department of Neurology, Hirslanden Klinik Zurich, 8032 Zurich, Switzerland; 5 Swiss Epilepsy Center (Klinik Lengg), 8008 Zurich, Switzerland; 6 Center for Molecular Medicine, Neuroimmunology Unit, Department of Clinical Neuroscience, Karolinska Institutet, 17177 Stockholm, Sweden; klara.asplund@ki.se (K.A.H.); mohsen.khademi@ki.se (M.K.); faiez.al.nimer@ki.se (F.A.N.); 7 Clinical Research Priority Program MS (CRPP) PrecisionMS, University of Zurich, 8006 Zurich, Switzerland; 8 Neuroimmunology Outpatient Clinic, Center for Multiple Sclerosis, Neurocenter Bellevue, 8001 Zurich, Switzerland; 9 Infectious Disease Serology and Immunology, Medica Medizinische Laboratorien Dr. F. Kaeppeli AG, 8032 Zurich, Switzerland; axel.regeniter@medica.ch

**Keywords:** cerebrospinal fluid, multiple sclerosis, intrathecal IgG production, measles virus, rubella virus, varicella zoster virus, MRZ reaction, intrathecal polyspecific antiviral antibody reaction, pleocytosis, blood CSF barrier function

## Abstract

Background: The presence of intrathecal total IgG production is a hallmark of cerebrospinal fluid (CSF) characteristics in multiple sclerosis (MS). Herein, we systematically analyze how the intensity (instead of mere presence) of intrathecal total IgG production relates to basic CSF parameters in MS. Methods: We retrospectively assessed clinical routine CSF findings from 390 therapy-naïve relapsing-remitting MS patients diagnosed according to 2017 revised McDonald criteria. The intensity of intrathecal total IgG synthesis according to Reiber’s formula was stratified and correlated to demographics, CSF white cell count (WCC), and diversity of MRZ reaction, defined as a polyspecific intrathecal production of IgG reactive against ≥2 of the 3 viruses; measles (M), rubella (R), and varicella zoster (Z) virus. Results: The higher intensity of intrathecal total IgG production significantly correlated with higher CSF WCC (Spearman’s ρ = 0.433, *p* < 0.001) and with the increasing presence and diversity of positive MRZ reaction (Spearman’s ρ = 0.600, *p* < 0.001). Female patients showed higher intensity of intrathecal total IgG production and higher prevalence of positive MRZ reaction than males. Conclusions: The intensity of intrathecal total IgG production correlates with the degree of CSF WCC and diversity of MRZ reaction in MS. As yet unidentified female sex-related factors increase the intensity and diversity of intrathecal IgG production in MS.

## 1. Introduction

The intrathecal synthesis of IgG in patients with multiple sclerosis (MS) has been considered the immunological hallmark of MS-typical cerebrospinal fluid (CSF) changes since the 1970s [1]. With the establishment of the intrathecal presence of oligoclonal bands (OCB), CSF has gained great importance in the diagnosis of MS and differential diagnosis of autoimmune diseases of the central nervous system (CNS) due to their high sensitivity. In addition, the discovery of a polyspecific humoral immune response against various viruses in MS patients, mainly against measles (M), rubella (R), and varicella zoster (Z) viruses, the so-called MRZ reaction (MRZR) was derived and shown to represent the most specific biomarker for MS to date, complementing the relatively moderate specificity of OCB [2,3].

Since the now decades-old studies that form the basis of our understanding of MS-typical CSF changes and biomarkers, the diagnostic criteria for MS have repeatedly been revised, probably causing heterogeneity of CSF findings in the analyzed patient cohorts, especially regarding the presence of a positive MRZ reaction [3]. In addition, information about the sex balance in previous cohorts and differences in CSF findings between men and women is scarce, although there is recently growing evidence reporting sex-related differences in CSF parameters of MS patients [4,5,6,7]. Recent data have also shown that women present more frequently with intrathecal total IgG synthesis, and we have recently described a higher frequency of positive MRZR in women independent of other genetic confounders such as human leukocyte antigen (HLA) status [4,6]. Interestingly, intrathecal IgG production has been associated with earlier conversion to definite MS and disability worsening in MS patients [8,9], and women develop MS earlier with higher clinical and radiological disease activity, but show less rapid progression, less gray matter atrophy and a better long-term outcome than men [10]. However, it is less clear whether intrathecal IgG production and other basic CSF biomarkers affect clinical outcomes differently in women and men with MS.

Different methods have been applied to detect intrathecal IgG synthesis over the years, with the IgG index [11], Reiber’s formula [12,13], and isoelectric focusing (IEF) coupled with immunodetection (immunoblotting or immunofixation) [14,15] being the most commonly used methods. IEF for the detection of CSF-specific OCB is considered the most sensitive method to detect the presence of intrathecal IgG synthesis, and it is only a qualitative measure and does not inform about the intensity of intrathecal IgG production [15,16]. The IgG index consists of the CSF/serum quotients for IgG and albumin (Q_IgG_/Q_Alb_) and is frequently used due to its simplicity but it is not reliable due to its linearity, especially in samples with severe blood CSF barrier (BCSFB) dysfunction [15,16]. Because molecules of different sizes that originate from the blood (e.g., IgG and albumin) are in a nonlinear relationship to each other, the IgG index has been replaced by Reiber’s hyperbolic formula, which allows for quantification of the intensity of intrathecal IgG synthesis [13,17].

To better understand whether and how the intensity (and not just the mere presence) of intrathecal total IgG synthesis relates to basic CSF parameters in MS, herein, we systematically investigate correlations of the intensity of intrathecal IgG synthesis according to Reiber’s formula with demographics, MRZ reaction, CSF white cell count (WCC), and BCSFB function, in therapy-naïve patients with relapsing-remitting MS (RRMS) from two different cohorts diagnosed according to current McDonald criteria. Special attention is paid to sex-specific differences in CSF parameters.

## 2. Materials and Methods

### 2.1. Patients

Lumbar puncture (LP) findings from 390 therapy-naïve RRMS patients from two centers, a Swiss cohort from the University Hospital Zurich (n = 252) and a Swedish cohort from the Karolinska Institutet Stockholm (n = 138), were retrospectively collected from chart records and anonymized. All RRMS patients from Zurich with LP between 2008 and 2022 and all RRMS patients from Stockholm with LP between 2009 and 2017 were included, only when the results from routine clinical practice regarding CSF WCC, albumin, and total IgG concentrations in serum and CSF, OCB, and MRZR were available. No other inclusion or exclusion factors were taken into account. All patients fulfilled the 2017 revised McDonald diagnostic criteria of MS [18]. Relapse was defined according to Thompson et al. [18], and patients were considered to be “in remission” if they had no new symptoms or contrast-enhancing MRI lesions for >30 days before LP.

### 2.2. CSF and Serum Parameters

All CSF parameters were determined in the clinical laboratories of the two centers mentioned above as part of routine clinical analysis. CSF WCC and cytological examinations of the CSF cells were routinely obtained after a standardized protocol following the recommendations of the German Society of CSF Diagnostics and Clinical Neurochemistry (DGLN e.V.) as previously described [19,20]. Pleocytosis was defined as a CSF WCC > 4/μL. Concentrations of albumin, total IgG, total IgA and total IgM in CSF, and serum of Swiss and Swedish patients were quantified by immunonephelometry and their ratios calculated (Q_Alb_, Q_IgG_, Q_IgA_ and Q_IgM_, respectively). Notably, IgA and IgM was only assessed in Swiss patients. The function of the BCSFB was assessed using Q_Alb_. The upper reference limit of Q_Alb_, Q_Lim_, was calculated in an age-dependent manner according to Reiber [21]. Dysfunction of the BCSFB was defined as Q_Alb_ > Q_Lim_. The intrathecal, locally synthesized amount of immunoglobulin in CSF was calculated as IgG_Loc_, IgA_Loc_, and/or IgM_Loc_. The relative intrathecally produced immunoglobulin fraction of the total CSF immunoglobulin level was calculated as IgG_IF_, IgA_IF_, and/or IgM_IF_. Respective formulas of Reiber were applied [13]. IgG_IF_, IgA_IF_, and/or IgM_IF_ > 0% indicated intrathecal synthesis of IgG, IgA, and/or IgM, respectively. OCBs were detected by isoelectric focusing on agarose gels and immunoblotting using IgG-specific antibodies and a semi-automated approach. OCB patterns were evaluated according to international consensus criteria [14] and samples were separated: “with CSF-specific OCB” or “without CSF-specific OCB”. OCBs were considered CSF-specific, if ≥2 additional bands were detected in CSF compared to serum. IgG antibodies against measles- (M), rubella- (R), and varicella zoster (Z) virus were analyzed with a fully automated ELISA processing as previously described [20]. The virus-specific CSF/serum antibody index (CAI) was calculated and CAI ≥ 1.5 indicated intrathecal synthesis of virus-specific antibodies. The results were separated based on the number of positive CAI: 0/3 MRZ-CAI positive = MRZR_0_ (i.e., M-, R- and Z-CAI < 1.5); 1/3 CAI positive = MRZR_1_ (i.e., 1 of M-, R- or Z-CAI ≥ 1.5); 2/3 CAI positive = MRZR_2_ (i.e., 2 of M-, R- or Z-CAI ≥ 1.5); and 3/3 CAI positive = MRZR_3_ (i.e., all 3 M-, R- and Z-CAI ≥ 1.5). Additionally, the mean number of antiviral antibody species was calculated as

(n(MRZR_1_) + 2 ∗ n(MRZR_2_) + 3 ∗ n(MRZR_3_))/n

according to Reiber [17]. MRZR was interpreted as positive, if polyclonal intrathecal production of antibodies against ≥2 of the 3 viruses measles (M), rubella (R), and zoster (Z), was detectable, i.e., if MRZR_2_ or MRZR_3_ was detectable.

### 2.3. Statistics

Differences in age and disease duration were compared with the Kruskal–Wallis test. Differences in the frequency of qualitative parameters were compared with either Fisher’s exact or Chi-squared test, depending on the sample size. Differences in mean values of quantitative parameters were tested for normal distribution with the Shapiro–Wilk test and calculated using the Mann–Whitney U test or *t*-test, accordingly. Correlation analysis was performed by using Spearman–Rho test.

## 3. Results

### 3.1. Demographic Data and Basic CSF Parameters

At the time of conducting the lumbar puncture, 62.3% of patients were in relapse and 37.7% were in remission (Supplementary Table S1). A total of 67.4% of patients were females. The median age of patients was 33.0 years and the median disease duration was one month. Pleocytosis was present in 51.9% of patients (mean WCC 7.6/μL). A total of 19.7% of patients had BCSFB dysfunction with a mean Q_Alb_ of 4.9 × 10^−3^, Q_IgG_ of 4.5, Q_IgA_ of 1.7, and Q_IgM_ of 0.8. According to Reiber’s formula [13], 61.0% of patients showed the intrathecal synthesis of IgG, 8.8% of IgA, and 21.2% of IgM. CSF-specific OCB was present in 87.7% of cases. M-CAI was positive in 35.9%, R-CAI in 42.1%, and Z-CAI in 41.8% of patients. Overall, 39.2% had a positive MRZR.

### 3.2. Correlation of Intrathecal IgG Synthesis with Positive MRZ Reaciton and Single Virus CAIs

**Figure 1:**
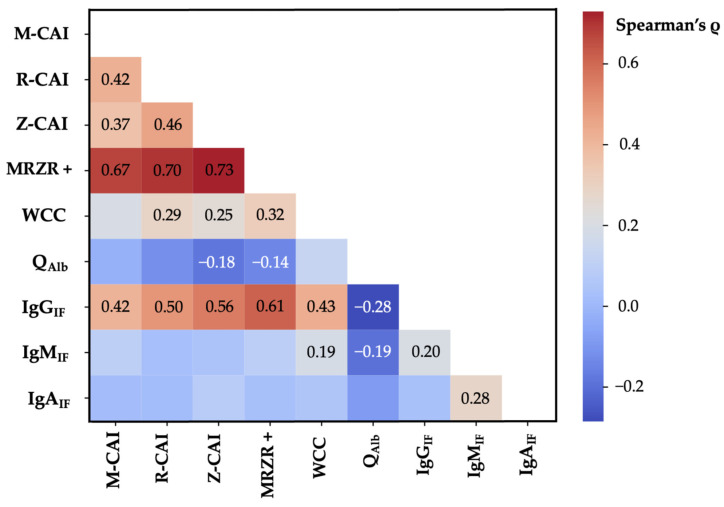
Heatmap with correlations between IgG_IF_ and (i) M-CAI values, (ii) R-CAI values, (iii) Z-CAI values, (iv) MRZ reaction positivity, (v) CSF white cell count, (vi) Q_Alb_, (vii) IgA_IF_, and (viii) IgM_IF_. Only Spearman’s ρ values for statistically significant correlations are shown. CAI: Virus-specific CSF/serum antibody index; IF: Intrathecal fraction; M: measles; MRZR +: Positive MRZ reaction; Q_Alb_: CSF/serum albumin ratio; R: Rubella; Z: Varizella zoster; WCC: CSF white cell count.

**Figure 2:**
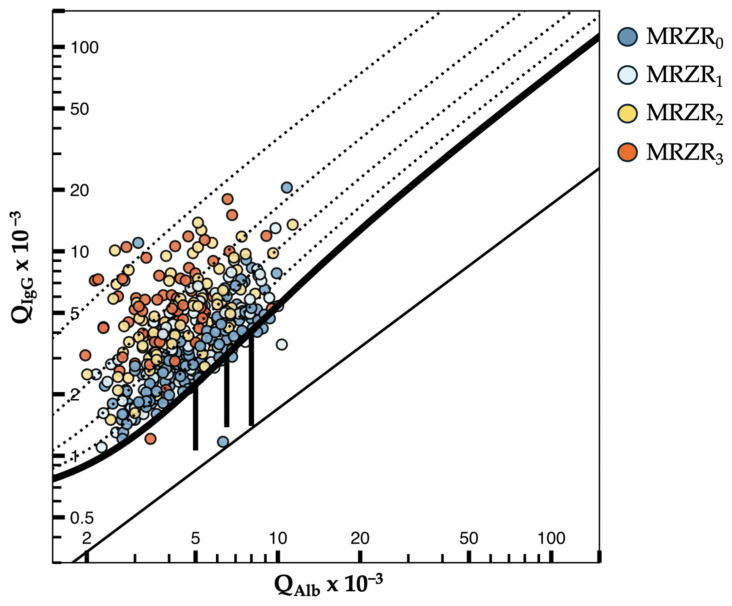
Degree of poly-specificity of MRZ reaction in MS patients in relation to intrathecal production of total IgG according to Reiber [13]. The Reiber diagram shows Q_IgG_ values in relation to Q_Alb_ values stratified according to samples with none (MRZR_0_ in dark blue), one (MRZR_1_ in light blue), two (MRZR_2_ in yellow) or three (MRZR_3_ in orange) different antibody species positive.

In a first step, patients were separated into six groups with intervals of increasing absolute amounts of IgG_Loc_ (Table 1). We found a strong correlation between mean IgG_Loc_ and mean number of antiviral antibody species (Spearman’s ρ = 0.600, *p* < 0.001), positive MRZR (Spearman’s ρ = 0.600, *p* < 0.001) and frequency of positive M-CAI (Spearman’s ρ = 0.398, *p* < 0.001), R-CAI (Spearman’s ρ = 0.471, *p* < 0.001), and Z-CAI (Spearman’s ρ = 0.469, *p* < 0.001), respectively (Table 1, Figure 1, Supplementary Table S2). Accordingly, the prevalence of MRZR_0_ and MRZR_1_ drops with increasing IgG_Loc_ while prevalence of MRZR_2_ and MRZR_3_ rises (Supplementary Table S3). The degree of poly-specificity of the MRZ reaction from MRZR_0_ over MRZR_1_ and MRZR_2_ to MRZR_3_ increases with stronger intrathecal total IgG production as detected in the Reiber diagram (Figure 2). This was not the case for total IgM and/or IgA production (Supplementary Figure S1). Furthermore, the frequency of female sex is strongly correlated to the amount of IgG_Loc_ (Spearman’s ρ = 0.289, *p* < 0.001), while there was no correlation between IgG_Loc_ and relapse/remission status, age at LP, or disease duration (Supplementary Tables S2 and S4). In addition, we confirmed significant differences in CSF WCC, pleocytosis, BCSFB dysfunction, Q_Alb_, and the intrathecal synthesis of total IgG between MS patients with positive and negative MRZR as previously reported (Supplementary Table S1, [7]).

In the second step, patients were separated into different groups with increasing relative fractions of IgG_IF_ (Table 2). We observed a highly significant correlation between mean IgG_IF_ and the amount of M-CAI (Spearman’s ρ = 0.417, *p* < 0.001), R-CAI (Spearman’s ρ = 0.484, *p* < 0.001), and Z-CAI (Spearman’s ρ = 0.562, *p* < 0.001) as well as the frequency of single virus-CAI positivity (Table 2, Figure 1, Supplementary Tables S2 and S5).

### 3.3. Correlation of Intrathecal IgG Synthesis with Other CSF Parameters

We observed a strong correlation between the fraction of IgG_IF_ and CSF WCC (Spearman’s ρ = 0.433, *p* < 0.001) and an inverse correlation of IgG_IF_ and Q_Alb_ (Spearman’s ρ = −0.284, *p* < 0.001) (Table 3, Supplementary Table S5).

With regard to IgG, there was a strong correlation between IgG_IF_ and IgG_CSF_ (Spearman’s ρ = 0.588, *p* < 0.001), but not between IgG_IF_ and IgG_Ser._ Furthermore, there was a correlation between IgG_IF_ and IgM_IF_ (Spearman’s ρ = 0.198, *p* = 0.002) as well as IgM_CSF_ (Spearman’s ρ = 0.153, *p* = 0.016), but not with IgM_Ser_, IgA_IF_, IgA_CSF_, or IgA_Ser_ (Supplementary Table S5).

We also analyzed nearly all correlations separately with regard to sex and relapse/remission status, respectively, and confirmed that they were independent of these factors (Supplementary Table S5). Exceptions comprised the correlation between IgG_IF_ and IgM_IF_ as well as IgM_CSF_, which was not significant in men alone, probably due to the small number of male patients (n = 92) analyzed, and the correlation between IgG_IF_ and IgM_IF_, which was positive in patients in remission.

### 3.4. Differences Between Male and Female MS Patients

As already indicated above, there is a strong correlation between the female sex and the amount of intrathecal IgG synthesis, i.e., IgG_Loc_. Therefore, we aimed to complement our work by analysis of sex differences in basic CSF parameters of MS patients.

The cohort included significantly more female than male patients (67.4% vs. 32.6%, *p* < 0.001), and female patients had a slightly longer median disease course at the time of LP (3.0 vs. 2.0 months, *p* < 0.001), but the groups did not differ in terms of relapse/remission status (*p* = 0.578) or age at LP (*p* = 0.916) (Table 4).

Pleocytosis tended to be more frequent in women than in men, but the effect was not statistically significant (55.3% vs. 44.9%, *p* = 0.066). Mean WCC was slightly higher in women (8.1 vs. 6.6, *p* = 0.043). Male patients had significantly more often BCSFB dysfunction (29.9% vs. 14.9%, *p* < 0.001) and higher mean Q_Alb_ (5.4 vs. 4.6, *p* < 0.001). Mean levels of IgG_Ser_ (10.9 g/L vs. 10.4 g/L, *p* < 0.001), IgG_CSF_ (50.5 mg/L vs. 41.8 mg/L, *p* = 0.014), and IgG_IF_ (25.8% vs. 11.4%, *p* < 0.001) as well as frequencies of intrathecal IgG synthesis according to Reiber (70.7% vs. 40.9%, *p* < 0.001) and CSF-specific OCB (90.1% vs. 82.7%, *p* < 0.001) were higher in women than men, while Q_IgG_ was not (Table 4 and Supplementary Table S6). Men had higher mean levels of IgA_CSF_ (4.0 mg/L vs. 3.1 mg/L, *p* = 0.030) and hence, higher Q_IgA_ (1.8 vs. 1.6, *p* = 0.029), but the groups did not differ in terms of IgA_Ser_, IgA_IF_, or frequency of intrathecal IgA synthesis according to Reiber (IgA_IF_). Mean IgM_Ser_ was higher in women (1.2 g/L vs. 1.1 g/L, *p* = 0.006), but there were no differences in IgM_CSF_, Q_IgM_, or frequency of intrathecal IgM synthesis according to Reiber (IgM_IF_). Frequency of positive M-CAI (40.3% vs. 26.8%, *p* = 0.010), R-CAI (47.9% vs. 29.9%, *p* = 0.001), and Z-CAI (45.6% vs. 33.9%, *p* = 0.029) as well as mean values of M-CAI (2.6 vs. 1.9, *p* = 0.021), R-CAI (2.9 vs. 1.6, *p* < 0.001), and Z-CAI (3.0 vs. 1.8, *p* = 0.003) were higher in women than in men (Supplementary Table S7).

Overall, a positive MRZ reaction was more frequent in female than male patients (45.2% vs. 26.8%, *p* = 0.001), especially with MRZR_3_ (19.8% vs. 8.7%, *p* = 0.005), while MRZR_0_ was more frequent in men (44.9% vs. 30.8%, *p* = 0.009) (Supplementary Table S7). Differences in frequency of MRZR_1_ and MRZR_2_ were not statistically significant.

## 4. Discussion

In this retrospective study, we have found that the intensity of intrathecal IgG production according to Reiber’s formula [12,13] in RRMS patients correlates with the degree of pleocytosis, the presence and diversity of intrathecally produced antiviral antibody species, i.e., presence and diversity of MRZ reaction, and female sex.

The relationship between intrathecal IgG synthesis and other parameters of the cellular and humoral immune response in the CSF of MS patients has been of interest for decades. However, all these studies over the last 40 years only investigated associations and/or correlations between intrathecal IgG production and only one additional basic CSF parameter such as the CSF WCC or MRZ reaction, using different definitions and calculation methods for intrathecal IgG production and different, outdated diagnostic criteria for MS. In addition, the cohorts analyzed in these studies differ in geographic origin (and therefore most probably in the distribution of HLA alleles increasing or reducing the probability of intrathecal IgG production) and/or patients’ sex, all of which may influence CSF parameters [3,4,6]. The interpretation of older studies is further complicated by varying cut-off values for the virus-specific CAI to define the positivity of MRZR [17]. To interpret our data vis à vis the existing studies, it is worthwhile to briefly review the history of reports about correlations between intrathecal total IgG synthesis and other CSF parameters.

Salmi and colleagues [22] were possibly the first to report a correlation between the intensity of intrathecal IgG synthesis and polyspecific antiviral antibody response in the CSF of patients diagnosed by the criteria of definite MS from 1965 [23]. To assess the intensity of intrathecal IgG synthesis in their cohort, an IgG index was calculated using linear regression for the mean CSF/serum antibody and albumin ratios, which was compared with 26 patients without intrathecal IgG synthesis [22]. This IgG index was strongly correlated to the number of intrathecally synthesized antiviral antibody species and the titer of the individual virus-specific antibody species. The antibody indices were calculated from the intrathecal antibody synthesis rates, where Log_2_ values of serum/CSF antibody ratios were used. Lauer and colleagues [24] analyzed mostly female MS patients diagnosed according to the MS criteria of 1983 [25], and calculated the intensity of intrathecal IgG synthesis according to Tourtellotte [26] as IgG synthesis rate per day [24]. This IgG synthesis rate correlated significantly with the CSF WCC. A year later, Felgenhauer and colleagues [27] confirmed the value of specific intrathecal IgG production against various neurotropic viruses in the diagnosis of MS, most of all against M, R, and Z, and therefore, shaped the term MRZR [27]. Reiber and Felgenhauer [28] went on to develop formulas to calculate the absolute amount of Ig_Loc_ as well as the relative intrathecally produced immunoglobulin Ig_IF_ and shaped the Reiber diagram, which is still considered the gold standard of CSF immunoglobulin interpretation today [13]. Reiber and Lange [29] finally increased the sensitivity of detecting virus-specific intrathecal antibody production with the combination of advanced enzyme immunoassay and the calculation of a ratio between the CSF/serum quotients for respective antigen-specific antibodies and total IgG [29]. The reference CAI value for specific intrathecal IgG synthesis was defined as ≥1.5. Reiber and colleagues [17] later analyzed a group of 267 MS patients, diagnosed according to the above-mentioned 1983 Poser criteria [25] with a cut-off >1.4 for CAI positivity. They demonstrated a correlation between the intensity of intrathecal IgG response and the amount as well as the diversity of antiviral antibody species in one of the biggest cohorts analyzed to date and interpreted this association as a consequence of an increasing number of B cell clones in the CNS [17]. In 2001, the McDonald diagnostic criteria of MS were developed [30] which were last revised in 2017 and are the gold standard of MS diagnosis to date [18]. More recently, a study reported a correlation between positive MRZR and intrathecal IgG synthesis, as measured by the kappa free light chain index and the relative proportion of intrathecally produced the kappa free light chain (KFLC) in MS patients diagnosed according to the 2017 revised McDonald criteria [31]. We have recently demonstrated the association between positive MRZR and intrathecal IgG synthesis in MS patients diagnosed according to 2017 revised McDonald criteria [7] and in line with this, Süsse and colleagues reported higher intrathecal KFLC production as a marker for the intensity of intrathecal IgG synthesis in MRZR-positive than in MRZR-negative patients [32].

Overall, our findings fit well with previous reports and summarize all these correlations and associations using state-of-the-art methods in MS patients diagnosed according to the latest diagnostic criteria [18]. We demonstrate a strong correlation between the intensity of the intrathecal IgG synthesis with the amount of CSF WCC, the number of intrathecally produced antiviral antibody species, the level of single virus-specific CAI values and the frequency of positive MRZR. Furthermore, there was a strong inverse correlation between the intrathecal total IgG synthesis and the frequency of MS patients with BCSFB dysfunction and respective Q_Alb_ values. We also observed a correlation between the intensity of intrathecal IgG synthesis with the frequency and intensity of intrathecal IgM synthesis. These correlations are independent of sex or relapse/remission status, which is particularly interesting because CSF basic parameters are known to differ in relapse and remission and predict future relapse risk [33].

The reason for the pronounced heterogeneity in the CSF of MS patients is not fully understood, but they are regarded to reflect brain pathology. Indeed, the differences are also evident at the histopathological level. Patients with lesions characterized by infiltration of T cells and macrophages (“pattern I”) frequently show intrathecal IgG synthesis and a positive MRZR, whereas these are rarely found in patients with additional evidence of antibody and complement deposition (“pattern II”) or distal oligodendrogliopathy (“pattern III”), who instead present BCSFB dysfunction with increased Q_Alb_ much more frequently [34]. An important role is attributed to immunogenetics, particularly the patients’ HLA alleles. Intrathecal IgG synthesis, determined by CSF-specific OCBs, the positive MRZR as well as the number of CSF B cells and plasma cells in MS patients are strongly associated with HLA-DRB1*15:01, while HLA-DRB1*04 is more frequently present in patients without CSF-specific OCBs [4,6]. Involvement of T cells and/or T-B cell interactions are among possible players that may be responsible for these HLA-associated differences [35]. Whether in addition to the composition of the inflammatory processes, other factors such as the localization of the inflammatory processes within the CNS, especially the proximity to the CSF space also play a role in the heterogeneity of CSF findings in MS patients remains to be clarified.

Furthermore, there are HLA-independent sex-related differences in the CSF changes in MS patients. In line with our work, previous data showed higher intrathecal total IgG production and higher prevalence of CSF-specific OCB in females than males [4]. In general, the humoral response in many autoimmune diseases appears to be stronger in female than in male patients [36]. This may be due to the influence of sex hormones, X chromosome-encoded immune factors, sex-related epigenetic dysregulations and/or sex-specific interactions with environmental factors. Interestingly, as opposed to intrathecal total IgG production, there was no difference in the prevalence of intrathecal total IgM or IgA production between female and male MS patients in our cohort. This indicates that sex-related factors do not influence intrathecal total IgM or IgA production in MS patients as much as intrathecal total IgG production. As previously discussed, the underlying immunopathophysiology of the above-discussed sex differences remains poorly understood, but sex-modulated differences in T and/or B cell functions are among possible mechanisms [6]. Interestingly, other immunological markers in CSF and blood also show sex-specific differences in MS, e.g., serum levels of macrophage-derived chemokine (MDC), epithe-lial-derived neutrophil-activating protein 78 (ENA-78), Eotaxin-2, a proliferation-inducing ligand (APRIL), TNF-related apoptosis-inducing ligand (TRAIL), and interleukin-20 (IL-20) [37,38], CSF levels of tumor necrosis factor-like weak inducer of apoptosis (TWEAK) [38], CD99 expression on CD4 T cells in the CSF [39], and circulating levels of neutrophil extracellular traps in serum [40].

Castellazzi and colleagues [5] aimed to analyze sex differences in a mixed cohort of neurological patients including MS patients, patients with other inflammatory neurological diseases, non-inflammatory neurological diseases, and undefined neurological diseases. Interestingly, the authors reported a higher frequency of intrathecal total IgG synthesis according to IgG index, Reiber diagram, and CSF-specific OCB, in line with our findings of a more frequent and a stronger intrathecal total IgG response in female MS patients, as well as higher Q_Alb_ in men [5]. The only difference between this study and our work is that they observed a higher amount of CSF IgG in men than women, which the authors interpreted as a generally higher occurrence of serum-derived proteins in the CSF. Since women with MS have a significantly higher frequency of intrathecal total IgG synthesis as well as a higher intensity of intrathecal total IgG production, higher CSF IgG levels in women appear more logical. The difference could be explained by the diversity of the patients in the Castellazzi work, as only about a quarter of the patients in the study had a diagnosis of MS. In a follow-up study, Castellazzi and colleagues analyzed sex differences in MS patients alone and confirmed higher Q_Alb_ in male patients, but they reported no significant differences in intrathecal IgG synthesis between male and female patients [41]. This report differs from various previous works mentioned above [4,6,7,36] and stands in contrast to their preliminary work [5], where the MS group was the biggest part of a cohort of neurological diseases, in which female patients had significantly higher frequency of intrathecal IgG synthesis. Nevertheless, our data—in line with previous data—suggest that CSF biomarkers in MS should be analyzed in a sex-stratified manner.

Future studies should ideally prospectively validate the findings of our study and should in addition explore the clinical utility of sex-stratified CSF analyses, especially with regard to prognosis of clinical outcomes and responses to immunomodulatory treatments. Future studies will probably also include novel revisions to the McDonald criteria for diagnosing multiple sclerosis (MS), which have only recently been announced [42] and which should enable an earlier diagnosis of MS, i.e., even in asymptomatic individuals and patients with formerly diagnosed clinically isolated syndrome including patients without CSF-specific OCBs. Whether the inclusion of such individuals, who generally show lower frequencies of intrathecal IgG production than definite MS patients according to the 2017 revised diagnostic criteria, will eventually result in lower frequencies of intrathecal IgG production in newly diagnosed MS cohorts, remains to be seen. Importantly, the 2024 revised diagnostic criteria have been announced to include intrathecal production of kappa free light chains (κ-FLC) as a criterion in addition to CSF-specific OCB and on par with dissemination in time since the CSF/serum κ-FLC index is considered to have similar diagnostic accuracy in MS as CSF-specific OCB [43]. Compared to the Reiber method for intrathecal IgG production, measures of intrathecal κ-FLC production appear to be a more sensitive marker not only for the presence but also for the intensity of intrathecal immunoglobulin synthesis, since they are quantitative markers as opposed to CSF-specific OCB [43]. Therefore, it will be interesting to see how the correlations and associations of intrathecal immunoglobulin production with other basic CSF parameters described above turn out when more sensitive κ-FLC measures are used. However, seven different methods to determine intrathecal κ-FLC production have been used among studies, i.e., the κ-FLC index, the κ-FLC quotient (Q_κ-FLC_), absolute CSF κ-FLC concentrations, or the relative intrathecally produced fraction of κ-FLC (κ-FLC_IF_) with four different hyperbolic functions described to calculate the latter [44]. In addition, cut-off values for these κ-FLC measures vary considerably across studies (e.g., κ-FLC index cut-off values ranging between 2.4 and 20), and most of the studies did not report how they were obtained. A meta-analysis found that a cut-off value for the κ-FLC index of 6.1 reliably discriminates CIS/MS patients from control subjects [43]. Further studies with larger cohorts need to show, which cut-off values for the κ-FLC index and other κ-FLC measures show the best diagnostic accuracy, and which of the κ-FLC measures is the most suitable in clinical routine practice. Another area of future interest is how the intensity of intrathecal IgG production is related to CSF and/or serum biomarkers of axonal injury, neurodegeneration and/or glial activation, such as neurofilament light chain, chitinase-3-like-1 and glial fibrillary acidic protein (GFAP) and to radiological parameters of neurodegeneration and/or acute inflammation, and whether sex differences play a role.

## 5. Conclusions

The intensity of intrathecal total IgG production correlates with the degree of pleocytosis, as well as the prevalence and diversity of intrathecally produced antiviral antibody species across the MRZR spectrum, which may indicate uniform pathophysiological mechanisms that lead to MS-typical network changes in the basic CSF findings. Since these alterations are significantly more prominent in female MS patients, research into MS-related CSF biomarkers should investigate sex differences as well as their causes and consequences in more detail.

## Figures and Tables

**Table 1:** Frequency of a positive MRZ reaction, mean number of antiviral antibody species, and frequency of females in MS patients stratified according to intervals of increasing mean IgG_Loc_ values.

IgG_Loc_ Intervals (mg/L)	n/Interval, n/N (%)	Positive MRZR, n/N (%)	Mean Number of Antiviral Antibody Species (SD)	Females, n/N (%)
0	152/390 (38.9%)	20/152 (13.2%)	0.5 (±0.8)	77/152 (50.7%)
>0–10	84/390 (21.5%)	24/84 (28.6%)	1.0 (±0.9)	61/84 (72.6%)
>10–20	60/390 (15.4%)	36/60 (60.0%)	1.7 (±1.0)	47/60 (78.3%)
>20–30	33/390 (8.5%)	21/33 (63.6%)	1.9 (±0.9)	27/33 (81.8%)
>30–40	17/390 (4.4%)	14/17 (82.4%)	2.2 (±0.9)	36/39 (82.4%)
>40	44/390 (11.3%)	38/44 (86.4%)	2.3 (±0.9)	37/44 (84.1%)

**Table 2:** Frequency of positive CAI for M, R, and Z (M-, R-, or Z-CAI ≥ 1.5) and median M-, R-, and Z-CAI values in MS patients stratified according to increasing IgG_IF_ values. The respective median CAI included all CAI values within the respective IgG_IF_ interval.

IgG_IF_ Intervals (%)	M-CAI ≥ 1.5 per Interval, n/N (%)	M-CAI Median [Q1, Q3]	M-CAI Range	R-CAI ≥ 1.5 per Interval, n/N (%)	R-CAI Median [Q1, Q3]	R-CAI Range	Z-CAI ≥ 1.5 per Interval, n/N (%)	Z-CAI Median [Q1, Q3]	Z-CAI Range
0	26/152 (17.1%)	0.8 [0.4, 1.0]	0–4.4	28/152 (18.4%)	0.8 [0.6, 1.2]	0–17.0	29/152 (19.1%)	0.9 [0.7, 1.2]	0–9.3
>0–20	20/70 (28.6%)	0.9 [0.3, 1.7]	0–10.1	22/70 (31.4%)	0.9 [0.5, 1.8]	0–8.3	20/70 (28.6%)	1.4 [0.7, 1.6]	0–7.0
>20–40	30/75 (40.0%)	1.1 [0.7, 2.4]	0–16.2	44/75 (58.7%)	1.8 [0.9, 3.5]	0–19.5	42/75 (56.0%)	2.0 [1.0, 3.3]	0–12.2
>40–60	35/55 (63.6%)	2.0 [1.0, 6.1]	0–68.6	39/55 (70.9%)	2.1 [1.2, 6.0]	0–16.7	37/55 (67.3%)	2.3 [1.3, 5.2]	0–34.6
>60	29/38 (76.3%)	4.9 [1.8, 7.4]	0–21.9	31/38 (81.6%)	5.2 [2.5–9.2]	0–21.2	35/38 (92.1%)	5.3 [2.6, 11.7]	0.9–44.2

**Table 3:** Frequencies of CSF pleocytosis, BCSFB dysfunction, and intrathecal synthesis of IgA and IgM according to Reiber [13] in MS patients stratified according to intervals of increasing IgG_IF_ values.

IgG_IF_ Intervals, %	Pleocytosis, n/N (%)	BCSFB Dysfunction, n/N (%)	IgA_IF_ > 0%, n/N (%)	IgM_IF_ > 0%, n/N (%)
0	49/151 (32.5%)	43/152 (28.3%)	10/101 (9.9%)	15/101 (14.9%)
>0–20	27/70 (38.6%)	9/70 (12.9%)	1/42 (2.4%)	7/42 (16.7%)
>20–40	51/75 (68.0%)	15/75 (20.0%)	2/48 (4.2%)	9/48 (18.8%)
>40–60	41/55 (74.5%)	7/55 (12.7%)	6/36 (16.7%)	12/36 (33.3%)
>60	34/38 (89.5%)	3/38 (7.9%)	3/23 (13.0%)	9/23 (39.1%)

**Table 4:** Comparison of basic CSF parameters in male and female MS patients.

Parameter	Overall	Male	Female	*p* Value
n/N (%)	390	127	263	**<0.001**
age at LP, median [Q1, Q3]	33.0 [28.0, 41.0]	32.0 [28.0, 42.0]	33.0 [28.0, 40.0]	0.916
in relapse, n/N (%)	243/390 (62.3%)	82/127 (64.6%)	161/263 (61.2%)	0.578
disease duration in months, median [Q1, Q3]	1.0 [0.0, 7.0]	2.0 [0.0, 22.0]	3.0 [0.0, 25.0]	**0.001**
pleocytosis, n/N (%)	203/389 (52.2%)	57/127(44.9%)	145/262 (55.3%)	0.066
WCC, mean cells/μL (±SD)	7.6 (±9.0)	6.6 (±8.0)	8.1 (±9.5)	**0.043**
BCSFBD, n/N (%)	77/390 (19.7%)	38/127 (29.9%)	39/262 (14.9%)	**<0.001**
Q_Alb_, mean (±SD)	4.9 (±1.8)	5.4 (±1.9)	4.6 (±1.7)	**<0.001**
IgG_IF_ > 0%, n/N (%)	238/390 (61.0%)	52/127 (40.9%)	186/263 (70.7%)	**<0.001**
IgA_IF_ > 0%, n/N (%)	22/250 (8.8%)	7/92 (7.6%)	15/158 (9.5%)	0.817
IgM_IF_ > 0%, n/N (%)	53/250 (21.2%)	20/92 (21.7%)	33/158 (20.9%)	0.874
IgG_IF_ in % if >0%, mean (±SD)	34.6 (±21.5)	27.9 (±16.4)	36.5 (±22.4)	**0.017**
IgA_IF_ in % if >0%, mean (±SD)	38.4 (±25.7)	37.2 (±24.3)	38.9 (±27.1)	0.837
IgM_IF_ in % if >0%, mean (±SD)	48.5 (±24.2)	46.2 (±30.6)	49.9 (±19.8)	0.833
CSF-specific OCB, n/N (%)	342/390 (87.7%)	105/127 (82.7%)	237/263 (90.1%)	**0.048**

Bold values indicate significant results.

## Data Availability

The anonymized data of this study are available from the corresponding author (I.J.), upon reasonable request.

## Supplementary Materials

The following supporting information can be downloaded at: https://www.mdpi.com/article/10.3390/antib13040102/s1, Table S1: Comparison of demographic features and basic CSF parameters between MS patients with positive and negative MRZ reaction; Table S2: Correlations of CSF parameters and demographic parameters with the intensity of intrathecal total IgG production in MS patients; Table S3: Frequency of antibody combinations in the MRZ reaction with none (MRZR_0_), one (MRZR_1_), two (MRZR_2_), or three (MRZR_3_) different antibody species positive, stratified according to intervals of increasing mean IgG_Loc_ values; Table S4: Frequencies of females and patients in remission, as well as median age at LP and median disease duration stratified according to intervals of increasing IgG_IF_ values; Table S5: Correlations of CSF parameters and demographic parameters with intensity of intrathecal total IgG production in MS patients; Table S6: Additional comparison of basic CSF parameters in male and female MS patients. Table S7: Comparison of single virus CAI and frequency of positive MRZ reaction in male and female MS patients; Figure S1: Degree of poly-specificity of MRZ reaction in MS patients in relation to intrathecal production of (a) total IgA and (b) total IgM according to Reiber [13].

## References

[1] Holmoy T. (2009). The discovery of oligoclonal bands: A 50-year anniversary. Eur. Neurol..

[2] Felgenhauer K., Reiber H. (1992). The diagnostic significance of antibody specificity indices in multiple sclerosis and herpes virus induced diseases of the nervous system. Clin. Investig..

[3] Jarius S., Eichhorn P., Franciotta D., Petereit H.F., Akman-Demir G., Wick M., Wildemann B. (2017). The MRZ reaction as a highly specific marker of multiple sclerosis: Re-evaluation and structured review of the literature. J. Neurol..

[4] Goris A., Pauwels I., Gustavsen M.W., van Son B., Hilven K., Bos S.D., Celius E.G., Berg-Hansen P., Aarseth J., Myhr K.M. (2015). Genetic variants are major determinants of CSF antibody levels in multiple sclerosis. Brain.

[5] Castellazzi M., Ferri C., Alfiero S., Lombardo I., Laudisi M., Tecilla G., Boni M., Pizzicotti S., Fainardi E., Bellini T. (2021). Sex-Related Differences in Cerebrospinal Fluid Plasma-Derived Proteins of Neurological Patients. Diagnostics.

[6] Neidhart S., Vlad B., Hilty M., Hogelin K.A., Ziegler M., Berenjeno-Correa E., Reichen I., Stridh P., Jelcic I., Khademi M. (2024). HLA Associations of Intrathecal IgG Production against Specific Viruses in Multiple Sclerosis. Ann. Neurol..

[7] Vlad B., Neidhart S., Hilty M., Asplund Hogelin K., Reichen I., Ziegler M., Khademi M., Lutterotti A., Regeniter A., Martin R. (2024). Intrathecal immune reactivity against Measles-, Rubella-, and Varicella Zoster viruses is associated with cerebrospinal fluid inflammation in multiple sclerosis. Mult. Scler..

[8] Dobson R., Ramagopalan S., Davis A., Giovannoni G. (2013). Cerebrospinal fluid oligoclonal bands in multiple sclerosis and clinically isolated syndromes: A meta-analysis of prevalence, prognosis and effect of latitude. J. Neurol. Neurosurg. Psychiatry.

[9] Gasperi C., Salmen A., Antony G., Bayas A., Heesen C., Kumpfel T., Linker R.A., Paul F., Stangel M., Tackenberg B. (2019). Association of Intrathecal Immunoglobulin G Synthesis with Disability Worsening in Multiple Sclerosis. JAMA Neurol..

[10] Angeloni B., Bigi R., Bellucci G., Mechelli R., Ballerini C., Romano C., Morena E., Pellicciari G., Reniè R., Rinaldi V. (2021). A Case of Double Standard: Sex Differences in Multiple Sclerosis Risk Factors. Int. J. Mol. Sci..

[11] Link H., Tibbling G. (1977). Principles of albumin and IgG analyses in neurological disorders. III. Evaluation of IgG synthesis within the central nervous system in multiple sclerosis. Scand. J. Clin. Lab. Invest..

[12] Reiber H. (1994). Flow rate of cerebrospinal fluid (CSF)—A concept common to normal blood-CSF barrier function and to dysfunction in neurological diseases. J. Neurol. Sci..

[13] Reiber H. (1998). Cerebrospinal fluid–physiology, analysis and interpretation of protein patterns for diagnosis of neurological diseases. Mult. Scler..

[14] Andersson M., Alvarez-Cermeno J., Bernardi G., Cogato I., Fredman P., Frederiksen J., Fredrikson S., Gallo P., Grimaldi L.M., Gronning M. (1994). Cerebrospinal fluid in the diagnosis of multiple sclerosis: A consensus report. J. Neurol. Neurosurg. Psychiatry.

[15] Freedman M.S., Thompson E.J., Deisenhammer F., Giovannoni G., Grimsley G., Keir G., Ohman S., Racke M.K., Sharief M., Sindic C.J. (2005). Recommended standard of cerebrospinal fluid analysis in the diagnosis of multiple sclerosis: A consensus statement. Arch. Neurol..

[16] McLean B.N., Luxton R.W., Thompson E.J. (1990). A study of immunoglobulin G in the cerebrospinal fluid of 1007 patients with suspected neurological disease using isoelectric focusing and the Log IgG-Index. A comparison and diagnostic applications. Brain.

[17] Reiber H., Ungefehr S., Jacobi C. (1998). The intrathecal, polyspecific and oligoclonal immune response in multiple sclerosis. Mult. Scler..

[18] Thompson A.J., Banwell B.L., Barkhof F., Carroll W.M., Coetzee T., Comi G., Correale J., Fazekas F., Filippi M., Freedman M.S. (2018). Diagnosis of multiple sclerosis: 2017 revisions of the McDonald criteria. Lancet Neurol..

[19] Tumani H., Petereit H.F., Gerritzen A., Gross C.C., Huss A., Isenmann S., Jesse S., Khalil M., Lewczuk P., Lewerenz J. (2020). S1 guidelines “lumbar puncture and cerebrospinal fluid analysis” (abridged and translated version). Neurol. Res. Pract..

[20] Vlad B., Reichen I., Neidhart S., Hilty M., Lekaditi D., Heuer C., Eisele A., Ziegler M., Reindl M., Lutterotti A. (2023). Basic CSF parameters and MRZ reaction help in differentiating MOG antibody-associated autoimmune disease versus multiple sclerosis. Front. Immunol..

[21] Reiber H., Otto M., Trendelenburg C., Wormek A. (2004). Reporting Cerebrospinal Fluid Data: Knowledge Base and Interpretation Software. Clin. Chem. Lab. Med..

[22] Salmi A., Reunanen M., Ilonen J., Panelius M. (1983). Intrathecal antibody synthesis to virus antigens in multiple sclerosis. Clin. Exp. Immunol..

[23] Schumacher G.A., Beebe G., Kibler R.F., Kurland L.T., Kurtzke J.F., McDowell F., Nagler B., Sibley W.A., Tourtellotte W.W., Willmon T.L. (1965). Problems of Experimental Trials of Therapy in Multiple Sclerosis: Report by the Panel on the Evaluation of Experimental Trials of Therapy in Multiple Sclerosis. Ann. N. Y. Acad. Sci..

[24] Lauer K. (1984). On the diagnostic value of different CSF investigations in multiple sclerosis. J. Neurol..

[25] Poser C.M., Paty D.W., Scheinberg L., McDonald W.I., Davis F.A., Ebers G.C., Johnson K.P., Sibley W.A., Silberberg D.H., Tourtellotte W.W. (1983). New diagnostic criteria for multiple sclerosis: Guidelines for research protocols. Ann. Neurol..

[26] Tourtellotte W. (1970). On cerebrospinal fluid immunoglobulin-G (IgG) quotients in multiple sclerosis and other diseases. A review and a new formula to estimate the amount of IgG synthesized per day by the central nervous system. J. Neurol. Sci..

[27] Felgenhauer K., Schadlich H.J., Nekic M., Ackermann R. (1985). Cerebrospinal fluid virus antibodies. A diagnostic indicator for multiple sclerosis?. J. Neurol. Sci..

[28] Reiber H., Felgenhauer K. (1987). Protein transfer at the blood cerebrospinal fluid barrier and the quantitation of the humoral immune response within the central nervous system. Clin. Chim. Acta.

[29] Reiber H., Lange P. (1991). Quantification of virus-specific antibodies in cerebrospinal fluid and serum: Sensitive and specific detection of antibody synthesis in brain. Clin. Chem..

[30] McDonald W.I., Compston A., Edan G., Goodkin D., Hartung H.P., Lublin F.D., McFarland H.F., Paty D.W., Polman C.H., Reingold S.C. (2001). Recommended diagnostic criteria for multiple sclerosis: Guidelines from the International Panel on the diagnosis of multiple sclerosis. Ann. Neurol..

[31] Huss A., Mojib-Yezdani F., Bachhuber F., Fangerau T., Lewerenz J., Otto M., Tumani H., Senel M. (2019). Association of cerebrospinal fluid kappa free light chains with the intrathecal polyspecific antiviral immune response in multiple sclerosis. Clin. Chim. Acta.

[32] Susse M., Reiber H., Grothe M., Petersmann A., Nauck M., Dressel A., Hannich M.J. (2020). Free light chain kappa and the polyspecific immune response in MS and CIS—Application of the hyperbolic reference range for most reliable data interpretation. J. Neuroimmunol..

[33] Stangel M., Fredrikson S., Meinl E., Petzold A., Stuve O., Tumani H. (2013). The utility of cerebrospinal fluid analysis in patients with multiple sclerosis. Nat. Rev. Neurol..

[34] Jarius S., Konig F.B., Metz I., Ruprecht K., Paul F., Bruck W., Wildemann B. (2017). Pattern II and pattern III MS are entities distinct from pattern I MS: Evidence from cerebrospinal fluid analysis. J. Neuroinflamm..

[35] Jelcic I., Al Nimer F., Wang J., Lentsch V., Planas R., Jelcic I., Madjovski A., Ruhrmann S., Faigle W., Frauenknecht K. (2018). Memory B Cells Activate Brain-Homing, Autoreactive CD4^+^ T Cells in Multiple Sclerosis. Cell.

[36] Klein S.L., Marriott I., Fish E.N. (2015). Sex-based differences in immune function and responses to vaccination. Trans. R. Soc. Trop. Med. Hyg..

[37] Jafarzadeh A., Ebrahimi H.A., Bagherzadeh S., Zarkesh F., Iranmanesh F., Najafzadeh A., Khosravimashizi A., Nemati M., Sabahi A., Hajghani H. (2014). Lower serum levels of Th2-related chemokine CCL22 in women patients with multiple sclerosis: A comparison between patients and healthy women. Inflammation.

[38] Berek K., Bauer A., Rudzki D., Auer M., Barket R., Zinganell A., Lerch M., Hofer L., Grams A., Poskaite P. (2023). Immune profiling in multiple sclerosis: A single-center study of 65 cytokines, chemokines, and related molecules in cerebrospinal fluid and serum. Front. Immunol..

[39] Winschel I., Willing A., Engler J.B., Walkenhorst M., Meurs N., Binkle-Ladisch L., Woo M.S., Pfeffer L.K., Sonner J.K., Borgmeyer U. (2024). Sex- and species-specific contribution of CD99 to T cell costimulation during multiple sclerosis. Biol. Sex. Differ..

[40] Tillack K., Naegele M., Haueis C., Schippling S., Wandinger K.P., Martin R., Sospedra M. (2013). Gender differences in circulating levels of neutrophil extracellular traps in serum of multiple sclerosis patients. J. Neuroimmunol..

[41] Castellazzi M., Ferri C., Tecilla G., Huss A., Crociani P., Desina G., Barbella G., Piola A., Permunian S., Senel M. (2022). The Sexual Dimorphism in Cerebrospinal Fluid Protein Content Does Not Affect Intrathecal IgG Synthesis in Multiple Sclerosis. J. Pers. Med..

[42] Montalban X. 2024 revisions of the McDonald criteria 2024. Proceedings of the European Committee for Treatment and Research in Multiple Sclerosis (ECTRIMS) Congress.

[43] Hegen H., Walde J., Berek K., Arrambide G., Gnanapavan S., Kaplan B., Khalil M., Saadeh R., Teunissen C., Tumani H. (2023). Cerebrospinal fluid kappa free light chains for the diagnosis of multiple sclerosis: A systematic review and meta-analysis. Mult. Scler..

[44] Hegen H., Arrambide G., Gnanapavan S., Kaplan B., Khalil M., Saadeh R., Teunissen C., Tumani H., Villar L.M., Willrich M.A.V. (2023). Cerebrospinal fluid kappa free light chains for the diagnosis of multiple sclerosis: A consensus statement. Mult. Scler..

