# Supplementary material for: Intensity of Intrathecal Total IgG Synthesis in Multiple Sclerosis Correlates with the Degree of Pleocytosis, Diversity of Intrathecal Antiviral Antibody Specificities, and Female Sex
Source: Antibodies (Basel). 2024 Dec 12;13(4):102. doi: 10.3390/antib13040102 (PMC11672439; doi:10.3390/antib13040102)
Supplement: Supplementary file 1 [file antibodies-13-00102-s001.zip › antibodies-3281037-supplementary.pdf]

SUPPLEMENTARY MATERIAL

Intensity of Intrathecal Total IgG Synthesis in Multiple Sclerosis Correlates with the Degree of Pleocytosis, Diversity of Intrathecal Antiviral Antibody Specificities, and Female Sex

Benjamin Vlad [1,2,3], Marc Hilty [1,2,4], Stephan Neidhart [1,2,5], Klara Asplund Högelin [6], Mario Ziegler [1], Mohsen Khademi [6], Andreas Lutterotti [1,2,4,7,8], Axel Regeniter [9], Roland Martin [1,2], Faiez Al Nimer [6] and Ilijas Jelcic [1,2,4,7,8,*]

[1] Neuroimmunology and Multiple Sclerosis Research Section, Department of Neurology, University Hospital Zurich, 8091 Zurich, Switzerland; benjamindaniel.vlad@med.uni-jena.de (B.V.); marc.hilty@hirslanden.ch (M.H.); stephan.neidhart@kliniklengg.ch (S.N.); mario.ziegler@usz.ch (M.Z.); andreas.lutterotti@bmg-swiss.ch (A.L.); roland.martin@uzh.ch (R.M.); ilijas.jelcic@uzh.ch (I.J.)
[2] Faculty of Medicine, University of Zurich, 8006 Zurich, Switzerland
[3] Department of Neurology, University Hospital Jena, 07749 Jena, Germany
[4] Department of Neurology, Hirslanden Klinik Zurich, 8032 Zurich, Switzerland
[5] Swiss Epilepsy Center (Klinik Lengg), 8008 Zurich, Switzerland
[6] Center for Molecular Medicine, Neuroimmunology Unit, Department of Clinical Neuroscience, Karolinska Institutet, 17177 Stockholm, Sweden; klara.asplund@ki.se (K.A.H.); mohsen.khademi@ki.se (M.K.); faiez.al.nimer@ki.se (F.A.N.)
[7] Clinical Research Priority Program MS (CRPP) Precision[MS], University of Zurich, 8006 Zurich, Switzerland
[8] Neuroimmunology Outpatient Clinic, Center for Multiple Sclerosis, Neurocenter Bellevue, 8001 Zurich, Switzerland
[9] Infectious Disease Serology and Immunology, Medica Medizinische Laboratorien Dr. F. Kaeppeli AG, 8032 Zurich, Switzerland; axel.regeniter@medica.ch
* Correspondence: ilijas.jelcic@uzh.ch

Supplementary Table S1. Comparison of demographic features and basic CSF parameters between MS patients with positive and negative MRZ reaction. Bold values indicate statistical significance (p <0.05).

Parameter	**Overall**	**Positive MRZ reaction**	**Negative MRZ reaction**	**p value**
n/N (%)	390/390 (100.0%)	153/390 (39.2%)	237/390 (60.8%)	**<0.001**
Age at LP, median [Q1, Q3]	33.0 [28.0, 41.0]	36.0 [28.0, 44.0]	32.0 [28.0, 38.0]	**0.020**
Female sex, n/N (%)	263/390 (67.4%)	119/153 (77.8%)	144/237 (60.8%)	**0.001**
Disease duration in months, median [Q1, Q3]	1.0 [0.0, 7.0]	1.3 [0.1, 10.0]	1.0 [0.0, 6.0]	0.408
In relapse, n/N (%)	243/390 (62.3%)	94/153 (61.4%)	149/237 (62.9%)	0.831
CSF WCC, mean cells/µl (±SD)	7.6 (±9.0)	10.1 (±11.8)	6.0 (±6.2)	**<0.001**
Pleocytosis, n/N (%)	202/389 (51.9%)	100/153 (65.4%)	102/236 (43.2%)	**<0.001**
BCSFB dysfunction, n/N (%)	77/390 (19.7%)	17/153 (11.1%)	60/237 (25.3%)	**0.001**
Q_{Alb} ($\times 10^{-3}$), mean (±SD)	4.9 (±1.8)	4.5 (±1.6)	5.1 (±1.9)	**0.006**
Q_{IgG}, mean (±SD)	4.5 (±2.6)	5.6 (±2.9)	3.7 (±2.1)	**<0.001**
Q_{IgA}, mean (±SD)	1.7 (±1.5)	1.5 (±1.0)	1.7 (±1.7)	0.428
Q_{IgM}, mean (±SD)	0.8 (±1.3)	0.8 (±1.5)	0.8 (±1.1)	0.577
Intrathecal synthesis of total IgG (Reiber), n/N (%)	238/390 (61.0%)	133/153 (86.9%)	105/237 (44.3%)	**<0.001**
Intrathecal synthesis of total IgA (Reiber), n/N (%)	22/250 (8.8%)	9/91 (9.9%)	13/159 (8.2%)	0.649
Intrathecal synthesis of total IgM (Reiber), n/N (%)	53/250 (21.2%)	22/91 (24.2%)	31/159 (19.5%)	0.423
CSF-specific OCB, n/N (%)	342/390 (87.7%)	150/153 (98.0%)	192/237 (81.0%)	**<0.001**

Q1, Q3 – first quartile and third quartile

SD – standard deviation

Supplementary Table S2. Correlations of CSF parameters and demographic parameters with intensity of intrathecal total IgG production in MS patients. Bold values indicate statistical significance (*p* <0.05).

Parameter	**Correlation with IgG_{Loc} (Spearman's ϱ, p value) in**									
	**All patients**		**Females**		**Males**		**Relapse**		**Remission**	
	**ϱ**	**p**	**ϱ**	**p**	**ϱ**	**p**	**ϱ**	**p**	**ϱ**	**p**
Mean number of antiviral antibody species	0.600	**<0.001**	0.582	**<0.001**	0.579	**<0.001**	0.642	**<0.001**	0.459	**<0.001**
Frequency of positive MRZR	0.600	**<0.001**	0.545	**<0.001**	0.479	**<0.001**	0.610	**<0.001**	0.535	**<0.001**
Frequency of positive M-CAI	0.398	**<0.001**	0.354	**<0.001**	0.415	**<0.001**	0.445	**<0.001**	0.329	**<0.001**
Frequency of positive R-CAI	0.471	**<0.001**	0.490	**<0.001**	0.329	**<0.001**	0.499	**<0.001**	0.424	**<0.001**
Frequency of positive Z-CAI	0.469	**<0.001**	0.458	**<0.001**	0.485	**<0.001**	0.501	**<0.001**	0.424	**<0.001**
Frequency of females	0.289	**<0.001**	-	-	-	-	0.312	**<0.001**	0.252	**0.002**
Frequency of patients in remission	0.013	0.805	-0.018	0.768	0.040	0.655	-	-	-	-
Age at LP	0.000	0.998	-0.020	0.748	0.023	0.801	0.048	0.461	-0.075	0.369
Disease duration in months	0.066	0.192	0.076	0.217	0.031	0.733	0.051	0.424	0.131	0.115

Supplementary Table S3. Frequency of antibody combinations in the MRZ reaction with none ($MRZR_0$), one ($MRZR_1$), two ($MRZR_2$) or three ($MRZR_3$) different antibody species positive, stratified according to intervals of increasing mean IgG_{Loc} values.

**IgG_{Loc} intervals (mg/l)**	**n/interval, n/N (%)**	**$MRZR_0$, n/N (%)**	**$MRZR_1$, n/N (%)**	**$MRZR_2$, n/N (%)**	**$MRZR_3$, n/N (%)**
0	152/390 (38.9%)	94/152 (61.8%)	38/152 (25.0%)	15/152 (9.9%)	5/152 (3.3%)
>0-10	84/390 (21.5%)	31/84 (36.9%)	29/84 (34.5%)	18/84 (20.2%)	6/84 (7.1%)
>10-20	60/390 (15.4%)	9/60 (15.0%)	15/60 (25.0%)	23/60 (38.3%)	13/60 (21.7%)
>20-30	33/390 (8.5%)	1/33 (3.0%)	11/33 (33.3%)	10/33 (30.3%)	11/33 (33.3%)
>30-40	17/390 (4.4%)	1/17 (5.9%)	2/17 (11.8%)	6/17 (35.3%)	8/17 (47.1%)
>40	44/390 (11.3%)	2/44 (4.5%)	4/44 (9.1%)	18/44 (41.9%)	20/44 (45.5%)

Supplementary Table S4. Frequencies of females and patients in remission, as well as median age at LP and median disease duration stratified according to intervals of increasing IgG_{IF} values.

**IgG_{Loc} intervals (mg/l)**	**in remission, n/N (%)**	**Age at LP, median [Q1, Q3]**	**Disease duration in months, median [Q1, Q3]**
0	53/152 (34.9%)	34.0 [28.0, 42.0]	1.0 [0.0, 6.0]
>0-10	39/84 (46.4%)	32.0 [28.0, 41.0]	1.0 [0.0, 6.8]
>10-20	19/60 (31.7%)	31.5 [25.5, 38.5]	1.2 [0.0, 11.0]
>20-30	12/33 (36.4%)	34.0 [28.0, 39.0]	1.5 [0.0, 10.0]
>30-40	6/17 (35.3%)	33.0 [30.0, 38.0]	1.0 [0.0, 3.0]
>40	18/44 (40.9%)	36.0 [30.0, 41.5]	1.5 [0.1, 11.6]

Supplementary Table S5. Correlations of CSF parameters and demographic parameters with intensity of intrathecal total IgG production in MS patients. Bold values indicate statistical significance (*p* <0.05).

Parameter	**Correlation with IgG_{IF} (Spearman's ρ, p value) in**									
	**All patients**		**females**		**males**		**relapse**		**remission**	
	**ρ**	**p**	**ρ**	**p**	**ρ**	**p**	**ρ**	**p**	**ρ**	**p**
Frequency of pleocytosis	0.405	**<0.001**	0.473	**<0.001**	0.209	**0.018**	0.416	**<0.001**	0.418	**<0.001**
Frequency of BCSFB dysfunction	-0.171	**0.001**	-0.123	**0.046**	-0.162	0.070	-0.214	**0.001**	-0.081	0.331
CSF WCC	0.433	**<0.001**	0.476	**<0.001**	0.304	**0.001**	0.448	**<0.001**	0.442	**<0.001**
Q_{Alb}	-0.284	**<0.001**	-0.260	**<0.001**	-0.194	**0.028**	-0.294	**<0.001**	-0.347	**<0.001**
IgG_{CSF}	0.588	**<0.001**	0.628	**<0.001**	0.444	**<0.001**	0.564	**<0.001**	0.626	**<0.001**
IgG_{Ser}	0.011	0.829	-0.028	0.650	-0.041	0.644	-0.050	0.440	0.130	0.115
IgM_{IF}	0.198	**0.002**	0.277	**<0.001**	0.079	0.454	0.160	**0.026**	0.265	0.046
IgM_{CSF}	0.153	**0.016**	0.169	0.033	0.062	0.559	0.100	0.167	0.286	**0.031**
IgM_{Ser}	0.049	0.440	-0.010	0.897	-0.023	0.830	0.035	0.630	0.072	0.597
IgA_{IF}	0.037	0.565	0.124	0.120	-0.152	0.148	-0.042	0.564	0.295	**0.026**
IgA_{CSF}	-0.119	0.060	-0.079	0.326	-0.147	0.163	-0.130	0.072	-0.085	0.527
IgA_{Ser}	-0.003	0.964	0.008	0.917	-0.028	0.788	-0.003	0.962	-0.033	0.807
Amount of M-CAI	0.417	**<0.001**	0.422	**<0.001**	0.380	**<0.001**	0.452	**<0.001**	0.356	**<0.001**
Amount of R-CAI	0.484	**<0.001**	0.530	**<0.001**	0.254	**0.004**	0.469	**<0.001**	0.508	**<0.001**
Amount of Z-CAI	0.562	**<0.001**	0.570	**<0.001**	0.526	**<0.001**	0.573	**<0.001**	0.555	**<0.001**

Supplementary Table S6. Additional comparison of basic CSF parameters in male and female MS patients. Bold values indicate statistical significance (p <0.05).

Parameter	Overall	Male	Female	p value
IgG_{Ser}, mean (±SD)	10.7 (±2.0)	10.4 (±1.9)	10.9 (±2.1)	**0.011**
IgG_{CSF}, mean (±SD)	47.7 (±29.2)	41.8 (±22.9)	50.5 (±31.4)	**0.014**
Q_{IgG}, mean (±SD)	4.5 (±2.6)	4.0 (±2.2)	4.6 (±2.8)	0.056
IgA_{Ser}, mean (±SD)	2.1 (±1.1)	2.2 (±1.4)	2.0 (±0.8)	0.703
IgA_{CSF}, mean (±SD)	3.5 (±4.3)	4.0 (±5.5)	3.1 (±3.3)	**0.030**
Q_{IgA}, mean (±SD)	1.7 (±1.5)	1.8 (±1.4)	1.6 (±1.5)	**0.029**
IgM_{Ser}, mean (±SD)	1.2 (±0.5)	1.1 (±0.5)	1.2 (±0.5)	**0.006**
IgM_{CSF}, mean (±SD)	0.8 (±1.3)	0.9 (±2.0)	0.8 (±0.8)	0.324
Q_{IgM}, mean (±SD)	0.8 (±1.3)	1.0 (±1.8)	0.6 (±0.7)	0.738

Supplementary Table S7. Comparison of single virus CAI and frequency of positive MRZ reaction in male and female MS patients. Bold values indicate statistical significance (*p* <0.05).

Parameter	Overall	Male	Female	p value
**Single virus-specific CAI**				
Positive M-CAI, n/N (%)	140/390 (35.9%)	34/127 (26.8%)	106/263 (40.3%)	**0.010**
Positive R-CAI, n/N (%)	164/390 (42.1%)	38/127 (29.9%)	126/263 (47.9%)	**0.001**
Positive Z-CAI, n/N (%)	163/390 (41.8%)	43/127 (33.9%)	120/263 (45.6%)	**0.029**
M-CAI, mean (±SD)	2.4 (±4.9)	1.9 (±3.3)	2.6 (±5.5)	**0.021**
R-CAI, mean (±SD)	2.5 (±3.5)	1.6 (±2.0)	2.9 (±3.9)	**<0.001**
Z-CAI, mean (±SD)	2.6 (±4.2)	1.8 (±1.9)	3.0 (±4.9)	**0.003**
M-CAI if >1.5, mean (±SD)	5.6 (±7.1)	5.4 (±4.8)	5.7 (±7.7)	0.273
R-CAI if >1.5, mean (±SD)	4.9 (±4.3)	3.7 (±2.4)	5.2 (±4.6)	0.068
Z-CAI if >1.5, mean (±SD)	5.1 (±5.6)	3.6 (±2.5)	5.6 (±6.3)	**0.049**
**MRZ reaction**				
$MRZR_0$, n/N (%)	138/390 (35.4%)	57/127 (44.9%)	81/263 (30.8%)	**0.009**
$MRZR_1$, n/N (%)	99/390 (25.4%)	36/127 (28.3%)	63/263 (24.0%)	0.385
$MRZR_2$, n/N (%)	90/390 (23.1%)	23/127 (18.1%)	67/263 (25.5%)	0.124
$MRZR_3$, n/N (%)	63/390 (16.2%)	11/127 (8.7%)	52/263 (19.8%)	**0.005**
Positive MRZ reaction, n/N (%)	153/390 (39.2%)	34/127 (26.8%)	119/263 (45.2%)	**0.001**

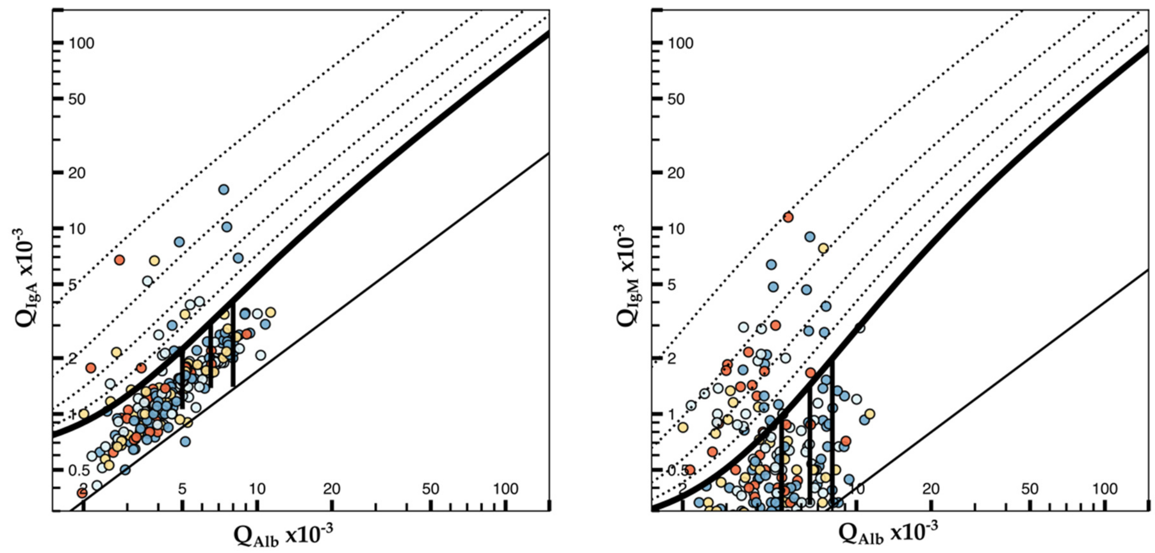

(a) **(b)**

Supplementary Figure S1. Degree of poly-specificity of MRZ reaction in MS patients in relation to intrathecal production of **(a)** total IgA and **(b)** total IgM according to Reiber [13]. The Reiber diagram shows Q_{IgG} values in relation to Q_{Alb} values stratified according to samples with none ($MRZR_0$ in dark blue), one ($MRZR_1$ in light blue), two ($MRZR_2$ in yellow) or three ($MRZR_3$ in red) different antibody species positive.
